# Perinatal depression and anxiety of primipara is higher than that of multipara in Japanese women

**DOI:** 10.1038/s41598-020-74088-8

**Published:** 2020-10-13

**Authors:** Yukako Nakamura, Takashi Okada, Mako Morikawa, Aya Yamauchi, Maya Sato, Masahiko Ando, Norio Ozaki

**Affiliations:** 1 Department of Psychiatry, Nagoya University Graduate School of Medicine, 65 Tsurumai-cho, Showa-ku, Nagoya, Aichi 466-8550 Japan; 2 Department of Child and Adolescent Psychiatry, Nagoya University Graduate School of Medicine, 65 Tsurumai-cho, Showa-ku, Nagoya, Aichi 466-8550 Japan; 3 Psychiatry/Child and Adolescent Psychiatry, Nagoya University Hospital, 65 Tsurumai-cho, Showa-ku, Nagoya, Aichi 466-8550 Japan; 4 Department of Advanced Medicine, Data Coordinating Center, Nagoya University Hospital, 65 Tsurumai-cho, Showa-ku, Nagoya, Aichi 466-8550 Japan

**Keywords:** Depression, Epidemiology

## Abstract

The proportion of women who experience a depressive state after delivery differs between primiparas and multiparas, so it is important to clarify the different factors related to depression between the two groups. In this study, we confirmed the differences in depressive states, the perinatal period, and social support between primiparas and multiparas, and clarified their characteristics. Data were extracted from a prospective cohort questionnaire survey conducted on pregnant women in Japan that included sociodemographic questions, the Edinburgh Postnatal Depression Scale, and the Japanese version of the Social Support Questionnaire. We carried out the chi-square test, Student’s *t*-test, and analysis of covariance to compare responses between primiparas and multiparas. A total of 1138 primiparas and 380 multiparas provided valid responses. We found that primiparas had higher rates of experiencing maternity blues and postpartum depression than multiparas. We also found that primiparas had higher anxiety scores than multiparas. Primiparas with postpartum depression perceived a lower number of persons available to provide social support than primiparas without postpartum depression. These findings suggest that it is important to provide pregnant women, especially for primiparas, with information that allows them to increase the number of people who can provide them with support.

## Introduction

Many mothers experience a large physical and psychosocial burden in the perinatal period, which can lead to a high risk of developing depression^1^. It is estimated that approximately 10–15% of mothers experience perinatal depression^2–5^, which has similar symptoms to major depressive disorder (MDD). In our previous study, we reported that 6.6% of pregnant women showed persistent depression during pregnancy to the postpartum period, and 10.4% showed depression for the first time after childbirth^6^.

The proportion of those who experience a depressed state after delivery differs between primiparas and multiparas^7–9^. In Japan, several studies have reported that the rate of women with depression after childbirth is higher for primiparas than multiparas^10–12^ and being a primipara has been proposed to be a risk factor for perinatal depression. In studies of non-Japanese subjects, it has been reported that being a multipara is a risk factor for postpartum depression (PPD)^13–15^. On a global scale, the prevalence of PPD differs by country and is greater than previously thought^16^. Therefore, it is important to clarify PPD characteristics and risk factors for each nation.

Mothers with maternity blues (MB) experience transient mood swings that usually subside within days after childbirth^17–19^. The prevalence rates of MB reported in the literature vary from 40 to 80%^20^. Based on a British study that used Stein’s Maternity Blues Scale (Stein’s Scale), 67% of postpartum women are diagnosed with MB^21^. However, studies in Japan have reported a prevalence of 15–35%^22–25^. Although MB is transitional, it is a highly prevalent mental health problem^26^. We previously reported that MB could be a risk factor for PPD^6^.

We also reported that social support has a protective function against a postpartum depressed state^27^. On the other hand, although support for mothers at high risk of PPD is useful, it has also been reported that support programs do not change mental health outcomes in mothers at low risk of PPD^28^. Social support is a limited resource, and it is important to identify groups that need more social support and groups that could experience a positive change with social support in order to make effective use of limited resources.

The differences in MB and social support by parity and their impact on PPD are unclear. In order to provide effective support for depressive maternal women, it is important to confirm the differences in social support and depressive states in the perinatal period between primiparas and multiparas and to clarify their characteristics. In this study, we focused our attention on parity and compared the proportion of primiparas and multiparas presenting with depressive state and MB in the perinatal period. In addition, we clarified the characteristics of Japanese primiparas considered at high risk of PPD.

## Results

**Figure 1:**
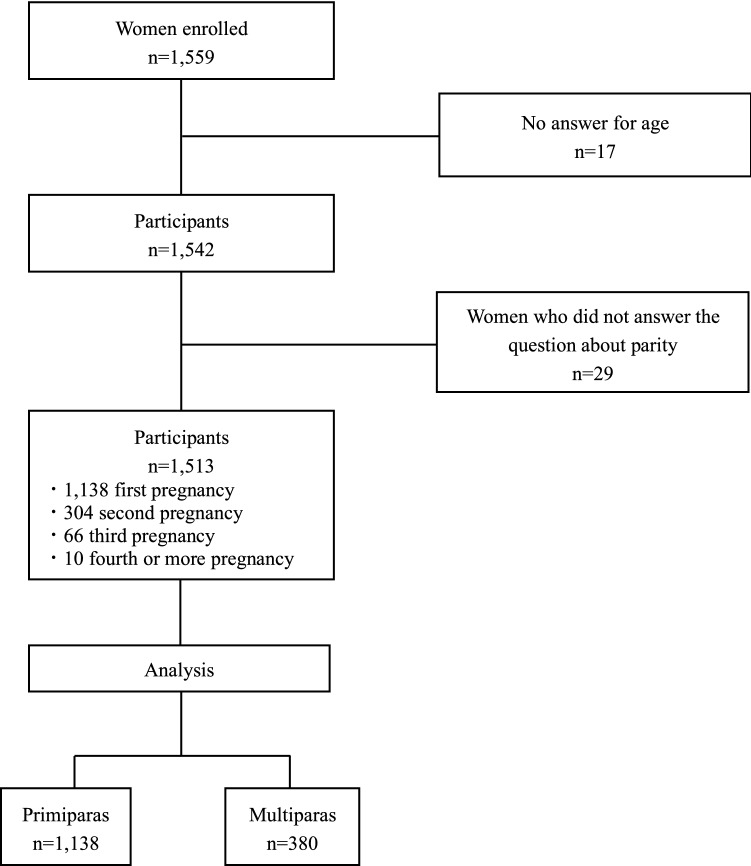
Flowchart of the recruitment process.

In total, 1559 women submitted valid questionnaire responses at around the 25th week of pregnancy. The average age of the participants was 32.4 ± 4.6 years (n = 1542). A total of 1138 women were recruited for this study during their first pregnancy, 304 women during their second pregnancy, 66 women during their third pregnancy, and 10 women during their fourth or more pregnancy. Forty-six women did not answer the question about age or parity. Therefore, this study included 1138 primiparas and 380 multiparas. A flowchart of the recruitment process is shown in Fig. 1. A comparison of the characteristics of primiparas and multiparas is shown in Table 1.

**Table 1:** Comparison of characteristics of primiparas and multiparas. IDDL, Inventory to Diagnose Depression, Lifetime version; SD, standard deviation.

	Primiparas (N = 1138)		Multiparas (N = 380)		Chi-square test
	n	%	n	%	*p*-value^a^
**IDDL**					
Negative	492	72.0	121	69.1	
Positive	191	28.0	54	30.9	0.450
**Presence of mental disease**					
Negative	493	83.7	135	85.4	
Positive	96	16.3	23	14.6	0.595
**Baby’s sex**					
Boy	318	50.8	85	52.1	
Girl	308	49.2	78	47.9	0.759

	Primiparas (N = 1138)		Multiparas (N = 380)		Student’s *t*-test
	n	Mean ± SD	n	Mean ± SD	*p*-value^b^
Age (years)	1133	32.0 ± 4.7	380	33.5 ± 4.1	3.0 × 10^–8^
Partner's age(years)	597	35.1 ± 6.0	161	36.0 ± 5.4	0.058
Education years	600	15.2 ± 2.0	161	14.9 ± 2.1	0.143
Baby’s weight	623	2995.1 ± 410.2	160	3001.7 ± 356.5	0.851

^a^Chi-square test between primiparas and multiparas.

^b^Student’s *t*-test between primiparas and multiparas.

Descriptive statistics were used to calculate means and percentages for participant characteristics and chi-square tests and Student’s *t*-tests were used to compare sociodemographic information based on parity (Table 1). Multiparas were significantly older than primiparas (*p* = 3.0 × 10^–8^, Student’s t-*t*est). Using a previously described method^29^, the history of MDD was calculated based on the Inventory to Diagnose Depression, Lifetime version (IDDL). There was no significant difference in the rate of prior history of MDD or presence of mental disease between primiparas and multiparas (*p* = 0.450 and 0.595, chi-square test). There was also no significant difference in years of education, partner’s age, baby’s weight (*p* = 0.143, 0.058 and 0.851, Student’s *t*-test), or baby’s sex (*p* = 0.759, chi-square test) among primiparas and multiparas. The main reason for missing values was differences in the time of information collection. The IDDL started to be conducted from April 2011, and data on years of education, presence or absence of mental disease, partner's age, and baby’s sex and weight started to be collected from June 2012.

The prevalence rates of PPD based on the Edinburgh Postnatal Depression Scale (EPDS) score at four time points (T1–T4) and MB based on Stein’s Scale at five time points (1, 2, 3, 4, and 5 days after delivery) for primiparas and multiparas are shown in Table 2. Means and standard deviations regarding responses to the EPDS were 24.3 ± 6.8 gestational weeks for T1, and 36.0 ± 0.9 gestational weeks for T2, 5.0 ± 0.6 days after delivery for T3, and 32.1 ± 5.2 days after delivery for T4. For the EPDS scores, the Bonferroni correction was performed by multiplying the number of test repetitions (four time points) by the *p*-value of the chi-square test, and for Stein’s Scale scores, the Bonferroni correction was performed by multiplying the number of test repetitions (five time points) by the *p*-value of the chi-square test (Table 2). We confirmed the difference between the non-depressive group and the PPD group using the chi-square test.

**Table 2:** Rate of PPD and MB in primiparas and multiparas.

	Primiparas		Multiparas		Chi-square test		
	n	%	n	%	*p*-value^c^		
**EPDS**^a^							
T1 score ≤ 8	915	81.0	306	81.8			
T1 score ≥ 9	214	19.0	68	18.2	1.000		
T2 score ≤ 8	867	85.2	293	86.4			
T2 score ≥ 9	151	14.8	46	13.6	1.000		
T4 score ≤ 8	790	77.5	291	83.9			
T4 score ≥ 9	229	22.5	56	16.1	0.048		
T5 score ≤ 8	794	76.9	300	86.2			
T5 score ≥ 9	238	23.1	48	13.8	9.0 × 10^–4^		
**Stein’s scale**^b^							
Day1 score ≤ 7	823	82.4	304	89.1			
Day1 score ≥ 8	178	17.6	37	10.9	0.016		
Day2 score ≤ 7	828	81.8	302	88.0			
Day2 score ≥ 8	184	18.2	41	12.0	0.037		
Day3 score ≤ 7	823	81.3	311	90.4			
Day3 score ≥ 8	189	18.7	33	9.6	4.2 × 10^–4^		
Day4 score ≤ 7	809	80.2	310	89.9			
Day4 score ≥ 8	200	19.8	35	10.1	2.1 × 10^–4^		
Day5 score ≤ 7	831	82.1	308	88.8			
Day5 score ≥ 8	181	17.9	39	11.2	0.019		

	Primiparas		Multiparas		Chi-square test	OR	CI (95%)
	n	%	n	%	*p*-value^d^		
**Depressive group**							
Non-depressive group	646	84.8	236	93.3			
Postpartum depressive group	116	15.2	17	6.7	0.002	2.09	1.33 – 3.31
**Maternity blues**							
Negative group	564	57.0	246	74.1			
Positive group	426	43.0	86	25.9	5.9 × 10^–8^	1.81	1.45 – 2.25

^a^Edinburgh Postnatal Depression Scale (EPDS), The cut-off point of EPDS is 8/9, with scores of 9 or higher used to screen for minor and major depressive episodes.

^b^Stein’s Maternity Blues Scale (Stein’s Scale), The cut-off point of Stein’s Scale is 7/8, with scores of 8 or higher indicating mood swings or maternity blues.

^c^Adjusted *p*-value of the Bonferroni correction, EPDS: Bonferroni correction was performed by multiplying the number of test repetitions (4 time points) from *p*-value of chi-square test, Maternity blues: Bonferroni correction was performed by multiplying the number of test repetitions (5 time points) from *p*-value of chi-square test.

^d^Adjusted *p*-value of the Bonferroni correction, Depressive group and Maternity blues: Bonferroni correction was performed multiplying the by number of test repetitions (2 factors) from *p*-value of chi-square test.

T1, around 25 weeks of gestation; T2, around 36 weeks of gestation; T3, 5 days after delivery; T4, 1 month after delivery; Day1, 1 day after delivery; Day2, 2 days after delivery; Day3, 3 days after delivery; Day4, 4 days after delivery; Day5, 5 days after delivery; PPD, postpartum depression; MB, maternity blues; OR, odds ratio; CI, confidence interval.

In primiparas, the ratio of PPD was high. Next, we confirmed the difference in MB between the negative group and the positive group using the chi-square test. In primiparas, the ratio of MB was high (Table 2).

We show the results regarding the association of PPD and MB with age in Table 3. The mean age did not significantly differ between the non-depressive and PPD groups or between the negative and positive of MB groups (*p* = 1.000 and 0.172, Student’s *t*-test). We confirmed that age had no influence on depression during pregnancy or the postpartum period, or on MB in this analysis. However, primiparas and multiparas showed a significant difference in age (Table 1); therefore, in our subsequent analyses (Tables 4, 5, 6) we chose a method that strictly excluded the influence of age. First, we performed the parallel test and confirmed that there was no interaction between the factor and the covariate. Next, we performed an analysis of covariance (ANCOVA) with age as a covariate.

**Table 3:** Association of PPD and MB with age.

	n	Mean (age)	± SD	Student’s *t*-test *p*-value^a^
**Depressive group**				
Non-depressive group	895	32.7	± 4.5	
Postpartum depressive group	138	32.7	± 4.2	1.000
**Maternity blues**				
Negative group	818	32.6	± 4.5	
Positive group	524	32.2	± 4.7	0.172

PPD, postpartum depression; MB, maternity blues; SD, standard deviation.

^a^Adjusted *p*-value of the Bonferroni correction, Depressive group and Maternity blues: Bonferroni correction was performed multiplying the by number of test repetitions (2 factors) from *p*-value of Student’s *t*-test.

**Table 4:** Comparison of EPDS factor scores between primiparas and multiparas from pregnancy to postpartum.

	T1					T2					T3					T4				
	Primiparas		Multiparas		ANCOVA^a^	Primiparas		Multiparas		ANCOVA ^a^	Primiparas		Multiparas		ANCOVA ^a^	Primiparas		Multiparas		ANCOVA ^a^
	N	Mean ± SD	N	Mean ± SD	*p*-value ^b^	N	Mean ± SD	N	Mean ± SD	*p*-value ^b^	N	Mean ± SD	N	Mean ± SD	*p*-value ^b^	N	Mean ± SD	N	Mean ± SD	*p*-value ^b^
Anxiety	1129	1.9 ± 1.7	375	1.5 ± 1.6	0.011	1018	1.6 ± 1.5	341	1.3 ± 1.5	0.062	1016	1.5 ± 1.6	347	1.0 ± 1.5	4.8 × 10 ^-5^	1031	1.6 ± 1.6	348	1.0 ± 1.4	9.2 × 10^–9^
Depression	1128	0.5 ± 1.0	378	0.6 ± 1.1	1.000	1017	0.5 ± 1.0	342	0.6 ± 1.0	1.000	1015	0.6 ± 1.0	347	0.6 ± 1.2	1.000	1029	0.5 ± 1.0	347	0.4 ± 0.9	1.000
Anhedonia	1129	0.3 ± 0.8	377	0.3 ± 0.9	1.000	1018	0.2 ± 0.7	340	0.2 ± 0.7	1.000	1018	0.4 ± 0.9	349	0.3 ± 0.9	1.000	1031	0.4 ± 0.9	348	0.3 ± 0.8	1.000

T1, around 25 weeks of gestation; T2, around 36 weeks of gestation; T3, 5 days after delivery; T4, 1 month after delivery; SD, standard deviation; ANCOVA, analysis of covariance.

^a^ In order to remove the effect of age, ANCOVA was performed with age as a covariate.

^b^ Adjusted *p*-value of the Bonferroni correction: Bonferroni correction was performed by multiplying the number of test repetitions (3 factors × 4 time points = 12) from *p*-value of ANCOVA.

**Table 5:** Comparison of J-SSQ scores in primiparas and multiparas.

	Primiparas		Multiparas		ANOCVA^a^
	n	Mean ± SD	n	Mean ± SD	*p*-value^b^
**T1 J-SSQ**					
Number of persons	1132	4.0 ± 2.4	378	3.5 ± 1.6	0.001
Satisfaction rating	1115	5.0 ± 1.2	369	4.8 ± 1.1	0.013
**T4 J-SSQ**					
Number of persons	792	4.1 ± 2.7	206	3.5 ± 1.6	0.019
Satisfaction rating	779	4.9 ± 1.3	203	4.6 ± 1.3	0.009

J-SSQ, the Japanese version of the Social Support Questionnaire; SD, standard deviation; ANCOVA, analysis of covariance; T1, around 25 weeks of gestation; T4, 1 month after delivery.

^a^In order to remove the effect of age, ANCOVA was performed with age as a covariate.

^b^Adjusted *p*-value of the Bonferroni correction: Bonferroni correction was performed by multiplying the number of test repetitions (2 factors × 2 time points = 4) from *p*-value of ANCOVA.

**Table 6:** J-SSQ scores in primiparas with and without PPD.

	Primiparas non-depressive group		Primiparas postpartum depressive group		ANCOVA^a^
	n	Mean ± SD	n	Mean ± SD	*p*-value^b^
**T1 J-SSQ**					
Number of persons	644	4.3 ± 2.5	114	3.9 ± 2.1	0.173
Satisfaction rating	638	5.0 ± 1.2	111	5.0 ± 1.1	1.000
**T4 J-SSQ**					
Number of persons	485	4.4 ± 3.1	87	3.5 ± 1.6	0.011
Satisfaction rating	477	4.9 ± 1.3	86	4.9 ± 1.1	1.000

J-SSQ, the Japanese version of the Social Support Questionnaire; PPD, postpartum depression; ANCOVA, analysis of covariance; SD, standard deviation; T1, around 25 weeks of gestation; T4, 1 month after delivery.

^a^In order to remove the effect of age, ANCOVA was performed with age as a covariate.

^b^Adjusted *p*-value of the Bonferroni correction: Bonferroni correction was performed by multiplying the number of test repetitions (2 factors × 2 time points = 4) from *p*-value of ANCOVA.

Table 4 shows the EPDS scores from pregnancy to the postpartum period. Primiparas had a significantly higher Anxiety score than did multiparas at T1, T3, and T4 (*p* = 0.011, 5.0 × 10^–5^ and 9.1 × 10^–9^, ANCOVA).

A comparison of score on the Japanese version of the Social Support Questionnaire (J-SSQ) between primiparas and multiparas is shown in Table 5. Primiparas had higher “Number of Persons” (NP) scores than did multiparas at T1 and T2, and higher “Satisfaction Rating” (SR) scores at T1. The main reason for missing values was differences in the time of information collection, as we started the evaluation of J-SSQ scores during early pregnancy in August 2004, and that of 1 month after birth in April 2006.

Table 6 shows the results of J-SSQ scores in primiparas in the PPD group and non-depressive group. The PPD group had significantly lower postpartum NP scores than the non-depressive group (*p* = 0.011, ANCOVA).

## Discussion

We found that primiparas had a higher rate of experiencing MB and higher rates of PPD compared with multiparas. The rate of Japanese women who experience MB is reported to be 15–35%^22–25^. According to the results of the present study, the percentage of MB in primiparas (43.0%) is significantly higher than that in multiparas (25.9%). In addition, the rate of primiparas (15.2%) who experience PPD is higher than that of multiparas (6.7%). These results are consistent with previous reports for Japanese women, indicating that being primipara is a risk factor for PPD^10–12^. We previously identified MB as a possible risk factor for PPD^6^, therefore, health care providers need to pay special attention to primiparas who present with MB.

There is a significant difference in average age between primiparas and multiparas, and there are reports that both being young^30^ and being old^31^ are risk factors for PPD. It has also been reported that becoming a mother at a young age is not itself a risk factor if it does not lead to a social disadvantage^32^. According to our results, it was parity, not age, that was associated with anxiety and PPD.

In this study, we found that primiparas had higher anxiety scores than multiparas, and in primiparas, the ratio of PPD was higher than in multiparas. Therefore, we need to be aware of anxiety in primiparas from early pregnancy. Primiparas probably have little child care experience and may lack self-confidence in their maternal role^26^. It has been reported that primiparas have lower scores for maternal self-efficacy in neonatal care than do multiparas^33^. Less experience and a lack of confidence are likely to lead to anxiety. Therefore, the high level of anxiety about childbirth and childcare that primiparas experience for the first time would be not a pathological response but a natural one. However, some evidence that pregnancy and postpartum anxiety affect child development has been presented^34^. For example, it has been reported that maternal anxiety during early pregnancy has long-term associations with the cognitive development of the child to be born^35^. In addition, higher rates of behavioral and emotional problems have been reported in children whose mothers experienced high levels of anxiety in late pregnancy^36^. Further, maternal anxiety during pregnancy and the postpartum period have been reported to predict socio-emotional development^37^ and may be associated with developmental delays in children^38^. Therefore, there is a need to cope with and reduce anxiety in primiparas. Increasing child care experience could be a promising way to prevent or mitigate PPD in primiparas^39^. Perinatal maternal anxiety exposure may lead to negative outcomes in child development, and more evidence is needed^40^.

When comparing primiparas and multiparas, the NP and SR subscale scores of the J-SSQ were higher in primiparas than in multiparas. Furthermore, it was revealed that in primiparas, the NP subscale score of the J-SSQ was significantly lower in the PPD group than in the non-depressive group. Studies on the quality of life of pregnant women have reported that being a primipara and having support from family and partner are associated with a better quality of life^41^. As noted in our previous study, social support can help prevent perinatal depression^27^. Moreover, a study of primiparas reported that most pregnant women recognize their partner, mother, or friends as the main provider of support^42^. Thus, informal support is very useful for the mental health of pregnant women, but many women in the perinatal period tend not to seek help in the face of mental disorders^43^. Therefore, it is important to provide pregnant women with information to help them increase the number of people who can provide them with support. In addition, it is important to inform pregnant women’s family and friends that providing support is useful for the mental health of pregnant women. We are currently disseminating results of our research on perinatal mental health to pregnant women in perinatal classes, and are trying to provide information to their family and friends via a booklet about mental health that they can take home and use. It will be necessary to evaluate the effectiveness of these activities in a future study. There are also reports that expert support is useful for mothers who are at high risk of PPD^44^. Professional support may need to be considered for primiparas at a suspected high risk of PPD, such as those with low NP and SR subscale scores, those with high anxiety, and those experienced MB.

This study had several limitations. First, a selection bias was possible because the target facilities were limited to four hospitals. In addition, we could not include pregnant women who did not participate in the perinatal class. Second, the psychiatric variables used in this study, such as anxiety, depression, and anhedonia, were evaluated based on a self-reported questionnaire, not on a diagnosis by a psychiatrist. Third, the factors examined in this study were limited, as we did not examine mode of delivery or traumatic birth; such factors should be investigated in the future. Fourth, we only followed the participants until 1 month after delivery and could not confirm their progress over a longer term. In addition, we did not confirm the effects of anxiety and PPD on children. Long-term follow-up, including an investigation of these effects on children, is therefore necessary. In this study, we compared primiparas and multiparas, but since these groups had different members, they were independent of each other. In the future, it will be important to consider differences, such as the timing of first and second births, in the same woman.

## Conclusion

The results of this study indicated that primiparas had higher rates of experiencing MB and higher rates of PPD than did multiparas. We also found that primiparas had higher anxiety scores since pregnancy than did multiparas, indicating that special attention needs to be paid to primiparas who present with MB. Primiparas had higher scores on the NP and SR subscales of the J-SSQ than did multiparas. However, when looking only at primiparas, the NP subscales score was lower in the PPD group than in the non-depressive group. From this result, it is considered that the number of supporters is more strongly associated with perinatal depression than is satisfaction with support. It is therefore important to provide pregnant women, especially primiparas, with information that increases the number of people who can provide them with support. In addition, professional support may need to be considered for primiparas with a suspected high risk of PPD, such as those with low NP and SR scores, high anxiety, and those who have experienced MB. The results of this study suggest that primiparas and multiparas have different characteristics, so it will be important to focus on parity in future studies.

## Methods

### Design

**Figure 2:**
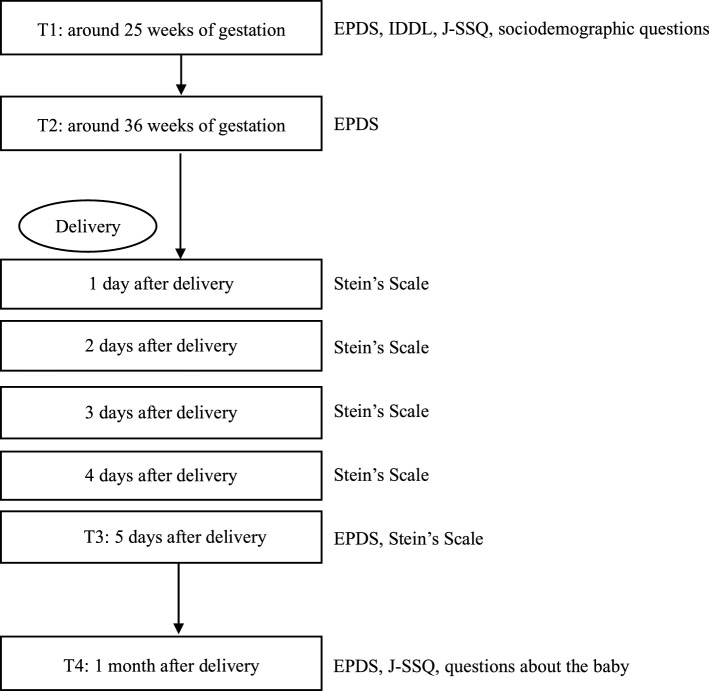
Flowchart of the study procedures. EPDS, Edinburgh Postnatal Depression Scale; IDDL, Inventory to Diagnose Depression, Lifetime version; J-SSQ, the Japanese version of the Social Support Questionnaire; Stein’s Scale, Stein’s Maternity Blues Scale.

The data in the present study were extracted from a prospective cohort study conducted in Nagoya, Japan from August 2004 to March 2020 that included a self-administered questionnaire survey. The questionnaire included the EPDS, IDDL, J-SSQ, Stein’s Scale, sociodemographic variables and questions about the baby’s sex and weight. Regarding the sociodemographic variables, we explored age, years of education, presence or absence of mental disease, partner’s age, and number of children. Among the sociodemographic questions and information on children, the age of the pregnant women and number of children were investigated from the beginning of the study in August 2004. We started collecting information about years of education, presence or absence of mental disease, partner’s age, and baby’s sex and weight in June 2012. The first questionnaire was completed in early pregnancy (T1: around 25 weeks of gestation) and included the EPDS, IDDL, J-SSQ, and sociodemographic questions. In late pregnancy (T2: around 36 weeks of gestation) and at 5 days after delivery (T3), the EPDS was completed again. On each of the first 5 days after delivery, Stein’s Scale was completed. At 1 month after delivery (T4), the EPDS, J-SSQ, and questions about the baby were completed. At 1 month after delivery, the completed questionnaire was returned by postal mail. A flowchart of the study procedure is shown in Fig. 2.

### Participants

Participants were recruited from one general hospital, two obstetrics and gynecology hospitals, and one university hospital. Pregnant women who were attending perinatal class that started before the 25th week of pregnancy and who met the following eligibility criteria were asked to participate: (1) pregnant female aged 20 years or older, and (2) ability to read and write Japanese. The age of adulthood in Japan is 20 years. Therefore, in our study, we excluded pregnant women under the age of 20 years because they were considered minors.

### Ethical considerations

A verbal and written explanation of the study was given to all participants, and written informed consent was obtained from all those who agreed to participate. The study protocol was approved by the Ethics Committee of the Nagoya University Graduate School of Medicine. All study methods met the Committee’s guidelines and regulations and all study protocols were in accordance with the 1964 Helsinki Declaration and its later amendments or comparable ethical standards.

### Measurements

#### EPDS

The EPDS is a self-administered questionnaire designed by Cox et al. in 1987 to screen for PPD^45^. The EPDS is comprised of 10 items and is scored on a four-point Likert scale (from 0 to 3), with total scores ranging from 0 to 30^45^. In our cohort study, we used the Japanese version of the EPDS, developed by Okano et al. in 1996^46^. Scores of ≥ 9 are used to screen for minor and major depressive episodes (sensitivity: 75% and 82%, respectively; specificity: 93% and 95%, respectively)^23,46^. In the present study, scores of ≥ 9 were used to identify women with depression. In the original version of the EPDS, the cut-off point is 12/13^45^, but in the non-English version, a cut-off point lower than 12/13 has been reported to be appropriate^47–50^. In this study, we used a cut-off point 8/9 for the Japanese version of the EPDS by Okano et al.^46^. The effectiveness of the EPDS as a screening scale for depression during pregnancy has also been reported^51^. The EPDS has three factors (“Anxiety”, “Depression”, and “Anhedonia”), and the factor structure has been demonstrated to be consistently stable throughout the peripartum period^52^.

We evaluated depressive symptoms using the EPDS during early pregnancy (around gestational week 25), during late pregnancy (around gestational week 36), at 5 days after delivery, and 1 month after birth. We categorized the participants into a “non-depressive group” or a “PPD group” based on EPDS score. Participants who scored under 8 points at the timepoints of approximately week 25, approximately week 36, and 1 month after birth were categorized into the non-depressive group. Participants who scored under 8 points at the timepoints of around week 25 and around week 36 but scored over 9 points at 1 month postpartum were categorized into the PPD group.

#### IDDL

To assess the history of MDD among mothers, we used the IDDL. The IDDL is a self-administered questionnaire used to assess the history of MDD. It is based on the third version of the Diagnostic and Statistical Manual of Mental Disorders^29^ and comprises 22 items scored from 0 to 4 (5-point Likert scale). If a symptom continues for more than 2 weeks, the respondent is considered as having that symptom. A score of 2–4 indicates the presence of a specific symptom for each item, except items 5 and 6, for which a score of 3–4 is used. The conditions regarding a history of MDD were as follows for items that last 2 weeks or more: (1) five or more items scored at the cut-off score or higher, and (2) these five or more items contain at least one item related to the two major symptoms. Uehara et al. reported the sensitivity and specificity of the Japanese version of the IDDL to be 83% and 97%, respectively^53^. We evaluated the history of MDD using the IDDL during early pregnancy (around week 25).

#### J-SSQ

The J-SSQ^54^ was used to measure social support among mothers in the present study. The J-SSQ has two factors, NP and SR^27^. NP is measured by the number subscale and reflects the average number of people the participant perceives as available to provide them with social support. SR is measured by the satisfaction subscale and reflects an individual’s average degree of satisfaction with the perceived support available in various situations^27^. The J-SSQ has confirmed reliability and validity in the pregnancy and postpartum periods^54^. We evaluated social support using the J-SSQ during early pregnancy (around week 25) and at 1 month after birth. We started the evaluation of J-SSQ during early pregnancy in August 2004, and that at 1 month after birth in April 2006.

#### Stein’s scale

Stein’s Scale was used to assess early postpartum mood^18^. This scale has 13 items with total score ranging from 0 to 26. The cut-off point is 7/8, with scores of 8 or higher indicating mood swings or MB^18^. The Japanese version of Stein’s Scale has proven validity and reliability for detecting MB in the early postpartum period^55^. Since MB is transitory in nature, it is recommended that self-evaluation be conducted on consecutive days after delivery^19^. Therefore, mothers in this study completed Stein’s Scale on each of the first 5 days after delivery. We categorized the participants into either a “negative group” or “positive group” based on Stein’s Scale scores. The negative group scored under 8 points on each of the 5 days after delivery. The positive group scored over 8 points on one or more days after delivery. We evaluated MB using the Stein’s Scale for 5 consecutive days (from the 1st to the 5th day after delivery).

### Statistical analyses

First, descriptive statistics based on the EPDS and J-SSQ were calculated. The listwise deletion method was used to handle missing data. All EPDS scores and J-SSQ subscale scores that were positively skewed were log-transformed in order to reduce the skewness. Then, the chi-square test, Student’s *t*-test and ANCOVA were conducted to compare primiparas and multiparas, using the adjusted *p*-value from the Bonferroni correction. We considered adjusted *p*-values of < 0.05 as significant.

## Data Availability

All the data supporting our findings are contained within the manuscript. Therefore, data sets are not shown. The raw data supporting the conclusions of this manuscript will be made available by the authors upon request.
